# An Integrated View of Aristolochic Acid Nephropathy: Update of the Literature

**DOI:** 10.3390/ijms18020297

**Published:** 2017-01-29

**Authors:** Inès Jadot, Anne-Emilie Declèves, Joëlle Nortier, Nathalie Caron

**Affiliations:** 1 Molecular Physiology Research Unit—URPhyM, Namur Research Institute for Life Sciences (NARILIS), University of Namur (UNamur), Namur 5000, Belgium; nathalie.caron@unamur.be; 2 Laboratory of Molecular Biology, Faculty of Medicine and Pharmacy, Research Institute for Health Sciences and Technology, University of Mons (UMons), Mons 7000, Belgium; anne-emilie.decleves@umons.ac.be; 3 Nephrology Department, Erasme Academic Hospital and Laboratory of Experimental Nephrology, Faculty of Medicine, Université Libre de Bruxelles (ULB), Brussels 1070, Belgium; joelle.nortier@erasme.ulb.ac.be

**Keywords:** aristolochic acids, herbal remedies, Balkan endemic nephropathy, renal interstitial fibrosis, urothelial carcinoma

## Abstract

The term “aristolochic acid nephropathy” (AAN) is used to include any form of toxic interstitial nephropathy that is caused either by ingestion of plants containing aristolochic acids (AA) as part of traditional phytotherapies (formerly known as “Chinese herbs nephropathy”), or by the environmental contaminants in food (Balkan endemic nephropathy). It is frequently associated with urothelial malignancies. Although products containing AA have been banned in most of countries, AAN cases remain regularly reported all over the world. Moreover, AAN incidence is probably highly underestimated given the presence of AA in traditional herbal remedies worldwide and the weak awareness of the disease. During these two past decades, animal models for AAN have been developed to investigate underlying molecular and cellular mechanisms involved in AAN pathogenesis. Indeed, a more-in-depth understanding of these processes is essential to develop therapeutic strategies aimed to reduce the global and underestimated burden of this disease. In this regard, our purpose was to build a broad overview of what is currently known about AAN. To achieve this goal, we aimed to summarize the latest data available about underlying pathophysiological mechanisms leading to AAN development with a particular emphasis on the imbalance between vasoactive factors as well as a focus on the vascular events often not considered in AAN.

## 1. Introduction

In the early 1990s, an epidemic of rapidly progressive tubulointerstitial nephritis was reported in Belgium in a cohort of young female patients. The onset of this the renal disease was rapidly associated with the intake of slimming pills containing Chinese herbs [1,2]. Among these herbs, the causative nephrotoxic agent was identified as aristolochic acid (AA) [3] and this renal disease is now worldwide recognized as aristolochic acid nephropathy (AAN). In addition, AA exposure has been also frequently associated with urothelial malignancies [4,5] and was classified as a human carcinogen class I by the World Health Organization International Agency for Research on Cancer in 2002 [6]. Since its discovery, more and more cases of AA intoxications have been reported all over the world and its incidence is probably much higher than initially thought [7], especially in Asia and in Balkan countries. Indeed, in Asian countries, traditional medicines are very popular and the complexity of the pharmacopoeia represents a high risk of AA-induced nephropathy due to the frequent use of *Aristolochia* species thereby increasing the potential risk of substitutions between botanical products [8,9,10,11]. In the Balkan regions, the exposure to AA has been admitted to be the causative agent responsible for the so-called Balkan endemic nephropathy (BEN) that occurs following ingestion of food prepared with flour derived from contaminated grains [12,13,14]. However, despite warnings from the Food and Drug Administration (FDA), the European Medicines Agency (EMA) and International Agency for Research on Cancer (IARC) regarding the safety of products containing AA, AAN cases remain frequently described all over the world [7,15].

## 2. The Facts—A Belgian Story of New Renal Disease

The initial report of the first recorded outbreak of AAN in Belgium in 1992 described simultaneous cases of women suffering from rapidly severe renal failure. Renal biopsy specimens typically showed progressive interstitial renal fibrosis with atrophy and loss of tubules predominantly located in the superficial cortex. These patients reached within a couple of months to end-stage renal disease (ESRD), requiring dialysis or transplantation [2]. It was found that all these patients had previously ingested pills containing Chinese herbs for slimming purposes from the same weight-loss clinical institute in Brussels [1], leading to the identification of a new type of nephropathy first called the “Chinese-herb nephropathy” (CHN) [2]. Very soon, further analyses of the slimming pills revealed that a constituent in the regimen, *Stephania tetrandra*, had been mistakenly substituted by *Aristolochia fangchi* because both herbs share the same common name in Pin Yin (respectively, Han Fang Ji and Guang Fang Ji) [3]. Therefore, *Aristolochia* was identified as the “bad boy” involved in the development of CHN. Indeed, *Aristolochia* species and more particularly one of their compounds, AA, had already been described by Mengs to be highly nephrotoxic [16]. AA involvement was first confirmed by the detection of AA-DNA adducts in renal tubular cells of biopsies from AA-intoxicated patients [5,17]. Their involvement was further confirmed by the positive correlation established between the cumulative ingested dose of AA and the progression of renal function deterioration [18]. Later, the presence of AA in a batch of so-called *Stephania tetrandra* was demonstrated by HPLC [19] and thereby validated the previous observations. All these findings led to rename this nephrotoxicity “aristolochic acid nephropathy” (AAN) [20].

## 3. Epidemiology of AAN and BEN: A True Link?

### 3.1. Aristolochic Acid Nephropathy

Despite the removal of *Aristolochia* species from the Belgian market, more than 100 cases of AAN were identified in Belgium in 1998 and 70% of these patients developed ESRD [1,7,15,21]. Furthermore, other cases of severe interstitial nephritis associated with progressive renal failure and urothelial malignancies consequently to the intake of Chinese herbs have been reported in other countries and are summarized in Table 1. In Spain, investigators reported a case of rapidly progressive renal failure in a man who had ingested a homemade mixture infusion containing mint and *Aristolochia pistolochia* [22]. In France, two similar cases of patient who had ingested slimming pills were described [23]. In 1999, Lord reported the first two cases of AAN in the United Kingdom in patients treated with Chinese herb remedies for eczema [24]. In Germany, a case a reversible Fanconi syndrome in a patient treated for hyper-uricaemia was observed [25]. AAN cases have also been described outside Europe; in the United States [26], Australia [27] and of course in Asia, precisely in China [28,29,30,31], Taiwan [8,9,32,33,34], Japan [35,36,37], Bangladesh [38], Korea [39] and Hong Kong [40]. Whereas AAN cases encountered in Western countries seem mostly to be due to uncontrolled use and/or false identification of medicinal herbs, generally considered as harmless by the general population [41], AAN cases in Asia are rather considered as the dramatic consequence of the complex pharmacopeia of herbal remedies used in traditional medicines. Indeed, AAN is even more crucial in Asian area, where people are confident that traditional Chinese herb medicine is more natural and safer than Western medicine [34]. In conclusion, all these reports taken together demonstrate that remedies containing AA have been and are still used in many regions of the world for various indications [42] and can also be easily bought via the Internet [38,42,43] or sometimes obtained in pharmacies and local markets without a doctor’s prescription [34]. However, high-quality epidemiologic data on the incidence and prevalence of AAN remain lacking, given the weak awareness of the disease. The risk of human exposure to AA remains therefore a global health problem with a potential growing incidence.

### 3.2. Balkan Endemic Nephropathy

The Balkan-endemic nephropathy (BEN) was first recognized in the late 1950s as a chronic renal disease affecting residents of rural farming villages located near tributaries of the Danube River. The geographic distribution of BEN has remained strikingly constant since its discovery. It is estimated that ~100,000 individuals are at risk of BEN while ~25,000 have currently developed the disease with the highest prevalence rates in Serbia, Bulgaria, Romania, Bosnia and Herzegovina and Croatia [44,45,46,47,48]. BEN is described as a familial clustering and slowly progressive kidney disease. The clinical signs and symptoms of BEN are non-specific and often remain latent for years even decades until a significant decline in function occurs [47,49,50]. After an initial asymptomatic stage, patients suffer from weakness and lassitude, mild lumbar pain and pallor of the skin [45,50]. At later stage (during the fifth or sixth decade of life), anemia is associated with a significant loss of renal function. Proteinuria of tubular type and specifically, β_2_-microglobulinuria is observed [46,48]. Histologically, BEN is characterized by tubular atrophy with extensive hypocellular fibrosis decreasing from the outer to the inner cortex of the kidney [45,46,50]. Moreover, kidney imaging reveals decrease in kidney size [45,46,48,50]. Finally, a major feature of BEN is its strong association with upper urothelial cancer (UUC) of the renal pelvis and ureter [47,50,51] that also constitutes the most common cause of death in BEN patients.

Over the past 60 years, numerous hypotheses on BEN causality have been considered as being linked to mycotoxins, heavy metals, viruses and trace element deficiencies. Environmental exposure to AA was first suggested as a potential cause of BEN by Ivic in 1969, but in 1993, more attention was provided to AA since similar renal histopathological features were shared between CHN and BEN [12]. Moreover, development of UUC in nearly 50% of CHN patients [5] constituted another important common similarity with BEN. Nowadays, environmental exposure to AA by BEN patients has been confirmed [52,53,54,55,56,57], especially through the detection of AA-DNA adducts and the hallmark A:T → T:A base transversion in renal cortical and urothelial malignant tissues [14,55,58].

The first speculation about the etiologic mechanism for chronic AA poisoning in BEN was also proposed by Ivic. He suggested that seeds from *Aristolochia*, which grow abundantly in wheat fields of endemic areas, were mixed with wheat grain during the harvesting process and therefore, AA might enter the human food chain through ingestion of bread prepared from flour derived from contaminated grain [53,56,59]. Recently, Pavlovic et al. [49] proposed another pathway by which AA could enter human bodies. Indeed, some crops grown in the fields where *Aristolochia clematitis* grows, senesces and decomposes during successive years might accumulate certain amounts of AA from the soil through root uptake and subsequently transfer it to other plant structures. In this regard, they showed that the roots of maize plant and cucumber were capable of absorbing AA confirming the possible involvement of naturally occurring root uptake in food chain contamination. Subsequently, AA were also identified in corn, wheat grain and soil samples collected from the endemic village of Kutles in Serbia providing the first direct evidence that food crops and soil are contaminated with AA in Balkan countries and thereby strengthening the intoxication pathway proposed earlier [60]. Finally, another recent study tested the hypothesis that AA could be translocated and bioaccumulated in food crops to cause chronic dietary poisoning [61]. To do so, analysis of the root uptake and transfer into the leaf of lettuce, the fruit of tomato and the bulb and leaf of spring onion grown in AA-contaminated soil was performed. The results of the study demonstrated that AA were resistant to the microbial activities of soil and were indeed translocated and bioaccumulated in food crops. AA were also described to be highly persistent to metabolism of plant cells and were present in food products for an extend period of time [61].

Although BEN and AAN share similar features, they differ in the clinical course. This observation can be easily explained by the fact that in BEN, AA are ingested in small doses via contaminated food while in AAN, AA are ingested in higher doses either intentionally or inadvertently [54,59]. Nevertheless, it is now clear that BEN is considered an environmental form of AAN [44,53,54,62].

## 4. Clinical Features of AAN

### 4.1. Nephrotoxicity

As mentioned earlier, numerous AAN cases have been described in the literature. Most of AAN patients were adults except one case report of AA intoxication in a 10-year-old boy [34]. Generally, renal failure was not suspected and was discovered by routine blood testing [15]. Besides few cases presenting with a Fanconi syndrome [25,34,39,63], most cases were characterized by renal failure. However, a hallmark of the disease was the rapid progression to an ESRD in 70% of AA-intoxicated patients [64]. Typically, moderate hypertension developed along with increased serum creatinine as well as a very severe anemia [7,64]. In addition, mild proteinuria and glycosuria were also reported. More precisely, elevated urinary excretion of five low molecular weight proteins (β2-microglobulin, cystatin C, Clara cell protein, retinal-binding protein and α1-microglobulin) confirmed that proteinuria derived from tubular origin [65]. Moreover, levels of urinary neutral endopeptidase (NEP), a 94 kDa ectoenzyme of the proximal tubule brush border, reflected the proportion of brush borders remaining intact at the apical side of proximal tubules and therefore constituted a reliable indicator of the severity of the renal disease. Indeed, urinary NEP was significantly decreased in AAN patient with moderate renal failure and almost undetectable in those with ESRD, indicating the loss of proximal tubule integrity [66]. Macroscopically, kidneys were shrunk, asymmetrical and irregular in cortical outline. Biopsies of intoxicated kidneys also revealed extensive paucicellular interstitial fibrosis with atrophy and loss of tubules starting from the peripheral cortex and progressing towards the deep cortex [1,2]. The glomeruli were described as spared even thought, in the later stage of the disease, they displayed a mild collapse of the capillaries and a wrinkling of the basement membrane. An interstitial inflammatory infiltration was observed in several renal biopsies, suggesting that an immunological process could be involved in the pathological mechanism, this later point being discussed in a following section.

### 4.2. Urothelial Malignancies

The strong correlation between AA intoxication and the presence of urothelial abnormalities has been intensively documented in clinical studies from AA-intoxicated patients [5,8,9,67] as well as in experimental models [16,68,69,70,71]. In 1994, Cosyns reported for the first time moderate atypia and atypical hyperplasia of the urothelium of native kidneys removed at the time of renal-allograft transplantation of Belgian AA-intoxicated women [12]. Indeed, significant proportion of the Belgian AAN patients developed upper tract urothelial carcinoma (UUC), and subsequently, bladder urothelial cancer [5,72]. Considering the high risk for urothelial malignancies related to AA intoxication, a prophylactic bilateral removal of the native kidneys and ureters was systematically proposed to AAN patients treated for dialysis or renal transplantation [5]. Further analysis also revealed that cumulative dose of ingested *Aristolochia* was a significant risk factor in developing urothelial carcinoma [5]. Moreover, it is greatly recognized that BEN is frequently associated with UUC corroborating with a mortality rate from rare urothelial cancer of the pelvis and ureters 55 times more frequent in the endemic region of Croatia where BEN incidence is really high than in the rest of the country [51]. Finally, in view of all these clinical and experimental data, the National Toxicology Program (NTP) classified AA among the highly carcinogenic substances [15]. Molecular mechanisms of AA-associated carcinogenicity are discussed below.

### 4.3. Diagnostic Criteria of AAN

The combination of interstitial nephropathy with urothelial malignancy strongly suggests the diagnostic of AAN. A consensus exists regarding the definition of diagnostic criteria [73]. AAN is certain in any individual who suffers from renal failure, in combination with at least two of the following three criteria: (i) a renal histology displaying interstitial fibrosis with a corticomedullary gradient; (ii) a history of consumption of herbal products which demonstrated the presence of AA; and (iii) the presence of AA-DNA adducts (or the specific A:T → T:A transversion in *p53* gene) in a kidney tissue sample or of a urothelial tumor. However, if only one of these criteria can be demonstrated, the diagnosis of AAN remains highly probable and should be investigated deeper. Whatever the case, the presence of either AA in herbal products ingested by patients, or of AA-DNA adducts in renal tissue, provides a serious hint to the diagnosis.

## 5. Properties of AA and Mechanisms of Nephrotoxicity and Carcinogenicity

Before the cluster outbreak of the so-called CHN in 1993 in Belgium, Mengs had already highlighted AA toxicity in animal models, especially their carcinogenic properties [16,68,69]. Since the identification of the Belgian cases, experimental in vitro and in vivo models have been developed [74,75] and studies have been therefore undertaken in order to understand underlying molecular and cellular mechanisms of AAN pathophysiology that are still matter of debate and require further investigations. Today, it is currently still unclear whether the nephrotoxic and carcinogenic effects of AA may be considered as closely related processes. In most cases, urothelial cancer seems to develop as a late complication in AAN patients since this carcinoma has always been detected in patients already suffering from ESRD. Nevertheless, only one case report described an invasive urothelial carcinoma after AA intoxication occurring without severe renal failure [76]. This observation may suggest that nephrotoxicity and carcinogenicity might be independent processes.

### 5.1. AA Structure-Activity Relationship

**Figure 1:**
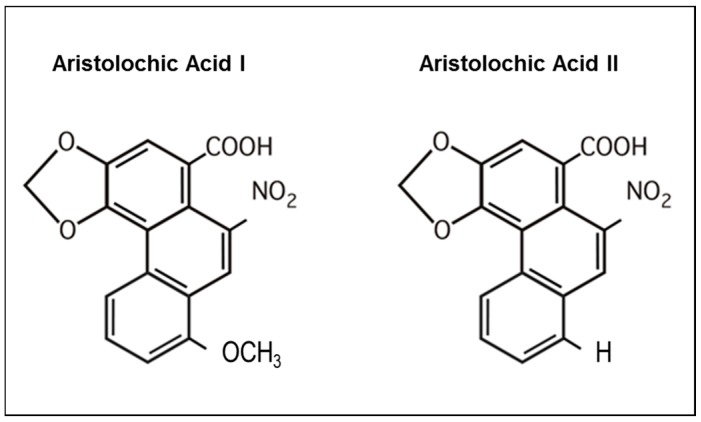
Molecular structure of aristolochic acids I and II. Formula of the major compounds of the *Aristolochia* species, aristolochic acid I (AAI) and aristolochic acid II (AAII).

AA is a generic name for a family of nitrophenanthrene carboxylic acids that are found in plants of the *Aristolochiaceae* family that includes almost 500 plants [15]. Most plants reported to contain AA belong to the genus *Aristolochia* or *Asarum* [44]. All parts of the plant including the roots, stems and leaves contain AA and are used in herbal preparation [42]. Chemically, AA is principally composed of a mixture of two metabolites, the 8-methoxy-6-nitro-phenanthro-(3,4-*d*)-1,3-dioxolo-5-carboxylic acid (AAI) and 6-nitro-phenanthro-(3,4-*d*)-1,3-dioxolo-5-carboxylic acid (AAII) (Figure 1). Few studies have been conducted in mice regarding structure–activity relationships and revealed that the carboxyl group at the 3-position is an absolute structural requirement for AAI transport and more particularly for its high affinity interaction with organic anion transporter (OAT) 1, 2 and 3, whereas the nitro group is only required by OAT1. Conversely, the *O*-methoxy group present at the 8-position in AAI is not requisite for transport [77] but seems to be rather a functional key determinant for AA-induced toxicity in mice [77,78]. In this regard, Shibutani conducted a study with C3H/He mice in order to differentiate the nephrotoxic and genotoxic properties of AAI and AAII [79]. It appeared that only AAI was capable of inducing nephrotoxicity as observed by tubular damage and development of interstitial fibrosis in AAI-treated mice whereas AAII did not show any nephrotoxic effect in the same mice. However, both AAI and AAII displayed genotoxic and carcinogenic effects as determined by their similar ability to form AA-DNA adducts in target tissues of intoxicated mice.

### 5.2. Exposition to AA

All known human exposures resulted from oral ingestion. AA are therefore absorbed from the gastrointestinal tract and distributed throughout the body. In this regard, Shibutani et al. described that after in vivo exposure to AA, DNA adducts derived from AAI and AAII metabolites are found in various rodent organs including bladder, stomach, intestine, liver, lung, spleen and of course, kidneys [79] thereby implying a systemic distribution. Interestingly, it has been described that once in blood circulation, AAI is able to bind to plasma proteins such as albumin, which will influence its distribution in the different body compartments as well as its further elimination since protein binding restrict AAI excretion through glomerular filtration [77]. After a metabolization step in the organism (see section below), AA metabolites (aristolactam I and aristolactam II) as well as AA-DNA and AA-RNA adducts have been found to be excreted in urine and in feces [44,80,81,82,83,84,85].

### 5.3. Role of OAT in AA-Induced Toxicity

In AAN, selective toxicity for proximal tubular epithelial cells (PTEC) suggests the involvement of specific molecular mechanism(s) that could be responsible for the accumulation of the toxin in these cells. In particular, proximal tubule plays a crucial role in the secretion and/or reabsorption of many compounds, including the elimination of xenobiotics or their metabolites, though several specific transporters. Therefore, PTEC may be considered as a potential target exposed to high concentrations of toxic metabolites. Regarding AA intoxication, several reports have proposed a role for the organic anion (OA) transporter (OAT) family in AA-mediated nephrotoxicity [77,86,87]. OAT family comprises a group of over 10 transmembrane proteins and are members of the solute carrier 22 (SLC22) subfamily [88,89,90]. OAT have been localized to almost all barrier epithelia of the body, as well as in the endothelium [89]. These transporters have been found to be expressed in many tissues including kidney, liver, intestine, choroid plexus, olfactory mucosa, brain, retina and placenta [89]. OAT are multiselective and may transport many classes of OA, including endogenous substances, such as urate and prostaglandins, but also exogenous molecules, such as antibiotics, nonsteroidal anti-inflammatory drugs, angiotensin-converting enzyme inhibitors as well as sulfate and glucuronide conjugates of drugs [90]. In the kidney, OAT substrates are small (molecular weight < 400 to 500 kDa) and water-soluble molecules [88,89] that display an ability to bind albumin [89,90]. Because of their albumin-binding capacity, these compounds are not filtered through the glomerulus so that they flow further through the peritubular capillaries. Renal secretion of these OA is therefore carried out in at least two steps: the first step is the transport from blood into PTEC through the basolateral membrane and the second one is the crossing of OA through the apical membrane of PTEC followed by the final excretion in the urine. In human kidneys, OAT1, OAT2 and OAT3 are both localized at the basolateral membrane of PTEC and mediate transport of substances from blood into the cytoplasm (influx transporters) [77,86,87,91,92]. OAT1 and OAT3 are predominantly expressed in the kidney whereas OAT2 is mainly expressed in the liver and weakly in the kidney [93]. Human OAT4 is expressed in the kidney and localized at the apical membrane of PTEC and is proposed to operate as an asymmetric organic anion exchanger that could play a role in anions reabsorption along with the simultaneous excretion of several anionic drugs and xenobiotics [86,87,91,93]. Both the four OATs mentioned above are sensitive to inhibition by the classical inhibitor, probenecid, albeit with different affinities [87,94].

**Figure 2:**
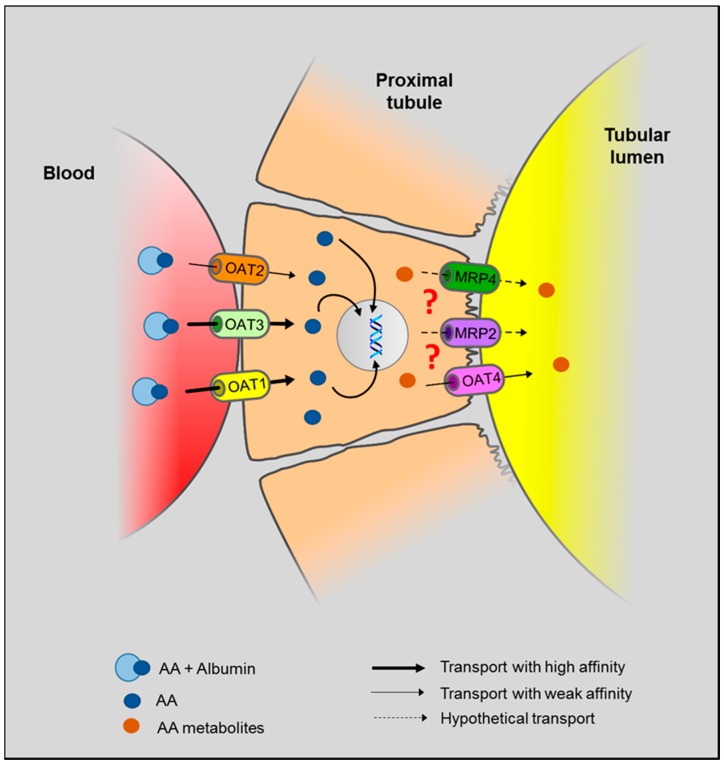
Proposed model for AA transport in the renal proximal tubule. In the bloodstream, AA have been found to bind to albumin. Once in peritubular capillaries, AA are transported through the basolateral membrane of PTEC via OAT1, OAT2 and OAT3. Then, AA metabolites bind to DNA (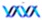) to from DNA adducts. Since AA metabolites are found in the urine, we hypothesize (?) that AA metabolites are secreted through the apical membrane in the proximal tubular lumen via OAT4. MRP2 and MRP4 could also be involved in the efflux of AA metabolites in the proximal tubule lumen. However, the majority of AA metabolites, the aristolactams, are trapped inside the cell due to the formation of specific DNA adducts.

Since AA display anionic properties [77,86,87] and have been found to bind albumin [77], OAT family has been considered as key players in AA-mediated toxicity. Support for this concept has been demonstrated for the first time by Bakhiya et al., who described AAI accumulation into HEK293 cells stably expressing human OAT1, -3 and -4 as well as the protective effect of probenecid treatment that reduced this accumulation in the same cells [91]. In addition, various other competition studies conducted in in vitro heterologous expression systems, also evaluated the interaction between AAI and OAT. It appeared that AAI is a high-affinity substrate for human and mouse OAT1 and OAT3 [77,86,91,95,96] but showed weak affinity for the human OAT4 [91]. These results suggest that the influx of AAI in PTEC through OAT1 and OAT3 is more important than their efflux through OAT4. This could therefore explain the toxic AAI accumulation in PTEC. Nevertheless, the exact role of OAT4 is still under investigation. As mentioned earlier, OAT4 proteins present asymmetric properties, so more evidences are needed to determine whether human OAT4 only function as an apical efflux transporter for AAI or may rather aggravate cellular injury by mediating additional AAI uptake from tubular fluid [87]. Moreover, once in the cells, AA rapidly bind to DNA to form DNA adducts so whether or not AA undergo apical transport or simply accumulate in the cell is still matter of debate. However, mRNA expression of OAT4 in human kidney cortex has been described to be very low compared to OAT1, -2 and -3, suggesting a minor involvement in drug-induced nephrotoxicity [87]. Finally, the role of OAT2 in AA handling remains unclear and its potential impact on AAN pathogenesis is still unknown. A hypothetical model of AA transport through PETC is available in Figure 2.

Studies on heterologous expression systems may have provide important clues about AA transport in PTEC, however it does not accurately reflect in vivo situation [87]. Only a few in vivo studies addressing AA transport in PTEC have been described in the literature [95,96,97]. In AA-treated mice, probenecid treatment has been found to decrease the renal distribution and the urinary excretion of AAI [95] but also to reduce tubular necrosis, lymphocytic infiltrate, tubular atrophy as well as fibrosis by blocking AA entry into PTEC as attested by the reduction of DNA-adducts formation [97]. In addition, *Oat1* and *Oat3* KO mice also displayed protection from experimental AA-induced nephropathy through reduction of AAI accumulation in PTEC [95]. Activity of these transporters together with metabolizing enzymes (see next section) could therefore be the main factors of AA-induced nephrotoxicity and tumorigenicity, allowing to consider OAT inhibition as a potential safe therapeutic pathway for AAN.

Finally, several other transport mechanisms than OAT have also been proposed regarding AA uptake into PTEC [77,95,97]. Since a time- and dose-dependent accumulation of AAI and AAII has been observed in control CHO-K1 transfected cells (that do not express OAT), it has been proposed that these compounds could enter the cells through simple diffusion [77]. Moreover, given that AAI can be detected in the urine and bile, they could also possibly undergo efflux via some other transporters than OAT4 such as multidrug resistance-associated protein (MRP) 2 or MRP4 expressed on the brush-border membranes in the kidney as well as MRP1, MRP2 and breast cancer protein at the canalicular membranes in the liver [95].

### 5.4. Metabolic Activation of AA—How That Works?

One of the common features of AAN and BEN is that not all individuals exposed to AA develop a nephropathy and/or a tumor. Besides differences in the accumulated dose of AA and the duration of AA intake [5,18], individual differences in the activities of the enzymes catalyzing the biotransformation of AA could be responsible for this discrepancy between the different levels of susceptibility [98]. In this regard, identification of the enzymes involved in AA metabolism is of particular relevance. A powerful tool to determine AA activation is to characterize and quantify DNA adducts formation and to identify which factor(s) could interfere with this process. Many in vitro studies conducted by Stiborova, Arlt and Schmeiser have addressed AA metabolism as well as biotransformation enzymes [99,100,101,102] and their major findings are summarized in a recent review [44].

**Figure 3:**
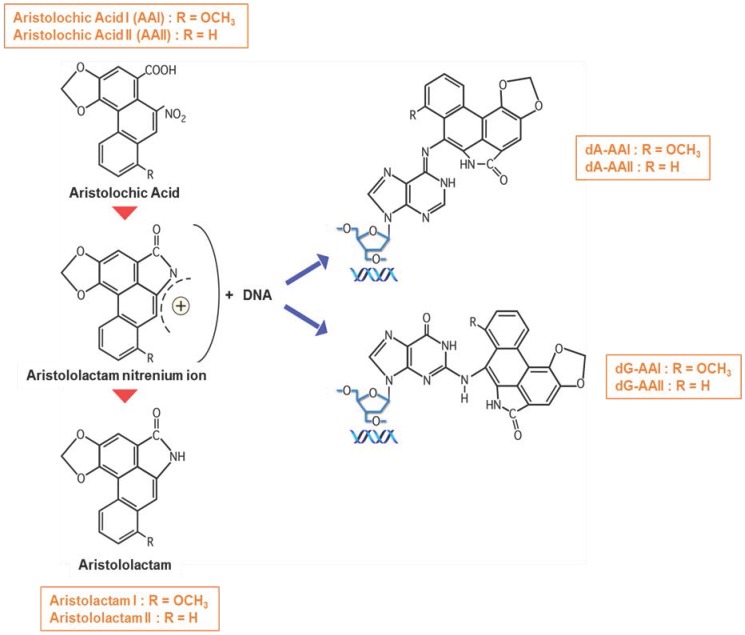
Metabolic activation of AA and DNA adduct formation. Aristolactam I and II are formed after the reductive metabolic activation mediated by hepatic and renal cytosolic NAD(P)H:quinone oxidoreductase (NQO1), hepatic microsomal cytochrome P450 (CYP) 1A1/2 and kidney microsomal NADPH:CYP reductase (POR). The electrophilic cyclic *N*-acylnitrenium ion has a delocalized positive charge able to bind to the exocyclic amino groups of purine bases and ultimately bind to DNA (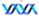) forming AA-DNA adducts.

The major activation pathway of AA involves nitroreduction to an electrophilic cyclic *N*-acylnitrenium ion with a delocalized positive charge able to bind to the exocyclic amino groups of purine bases and ultimately to DNA thereby leading to formation of AA-DNA adducts [55,103,104,105] (Figure 3). Presence of such AA-DNA adducts in renal tissue of patients may be considered as prominent biomarker in AAN diagnosis and has been demonstrated to persist during decades after AA exposure [15,17,62,106]. Biomonitoring of AA-DNA adducts in fresh frozen human renal tissues but also in formalin-fixed and paraffin-embedded samples as well as in exfoliated urinary cells can be achieved via ultra-performance liquid chromatography/ion-trap mass spectrometry, therefore providing a sensitive and specific method for establishing exposure to AA [59,107].

The nitroreduction of AA therefore constitutes a crucial step in AA-induced toxicity. In this regard, mammalian cells express a repertoire of enzymes able to ensure nitroreduction [98]. Among these, several have been shown to be involved in AA metabolization. The most important human, mouse and rat enzyme activating AAI in hepatic and renal cytosolic subcellular fraction is NAD(P)H:quinone oxidoreductase (NQO1) [84]. Hepatic microsomal cytochrome P450 (CYP) 1A1/2 and kidney microsomal NADPH:CYP reductase (POR) have also been shown to allow reduction of AAI [105]. In addition, genetic polymorphisms, drugs, smoking habit as well as environmental chemicals have been identified as important factors affecting the expression levels and activities of these enzymes and therefore could explain individual variations of susceptibility to AA toxicity [7].

Recently, it has been proposed that, in addition to interfere with DNA, AA could also modify cytoplasmic RNA and that the AA-induced insult to RNA might also contribute to AA-related toxicity. In this regard, Leung and Chan [108] first described that AA forms covalently bonded RNA adducts both in vitro and in vivo. Particularly, AA have been found to modify guanosine at significantly higher frequencies than adenosine suggesting that guanosine is a major target of AA and that guanosine adducts might constitute a critical factor in AA-induced nephrotoxicity and carcinogenicity.

### 5.5. Pathogenesis of AAN—What Do We Know from Experimental Studies?

#### 5.5.1. A Biphasic Evolution

**Figure 4:**
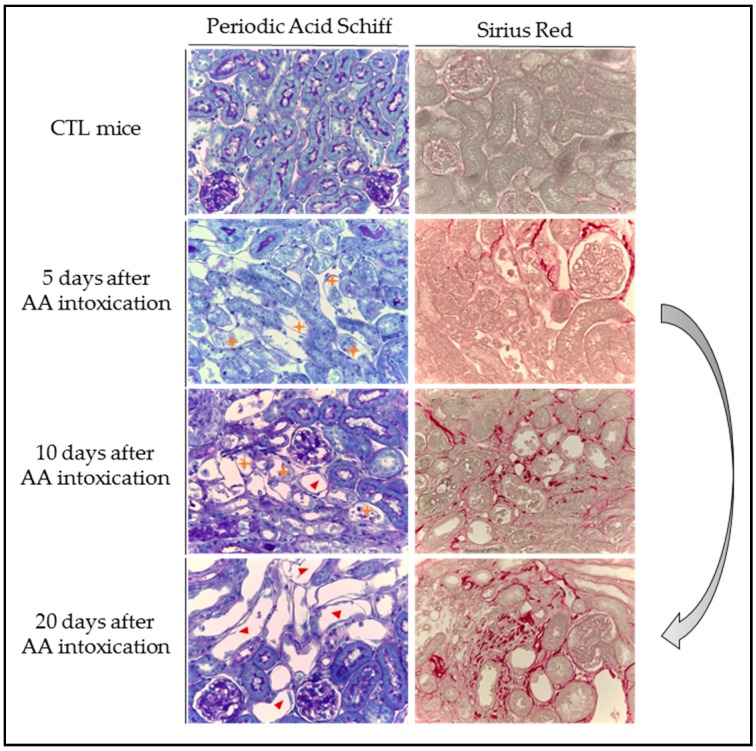
Evolution of structural abnormalities and collagen accumulation in experimental aristolochic acid nephropathy. Representative photographs of hemalun, Luxol fast blue and Periodic Acid Schiff stained kidney sections and Sirius Red stained kidney sections (400×) from CTL mice and mice intoxicated with AA (aristolochic acid I, Sigma-Aldrich, St. Louis, MO, USA) during four consecutive days. Mice were sacrificed 5, 10 and 20 days after first day of AA treatment. Necrotic tubules (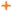) with cell debris in tubular lumens are visible in mice treated with AA at Days 5 and 10 and cystic tubules (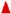) are visible in mice at Days 10 and 20. Collagen I and III, highlighted by Sirius Red staining, accumulate in the interstitium of the kidney of AA-treated mice from Day 10 and even more at Day 20 reflecting the progression to CKD.

In 2005, Lebeau et al. [109] investigated in detail the time course of structural and functional impairments of the proximal tubule in a rat model of AAN where AAN was induced by daily subcutaneous injections of AA for 5 or 35 days. A biphasic evolution of renal function and morphological alterations was described. Early proximal tubule dysfunction and structural abnormalities occurred within the first 3 days of the protocol. Indeed, necrosis of proximal tubular epithelial cells (PTEC) (Figure 4) was observed along with an increase of biochemical parameters such as tubular proteinuria, *N*-acetyl-β-d-glucosaminidase (NAG), α-glutathione *S*-transferase (α-GST), leucine aminopeptidase (LAP) and neutral endopeptidase (NEP) enzymuria. These early events were related to the formation of AA-DNA adducts that are detected as early as Day 1 of the protocol and reached a steady-state at Day 2, thereafter remaining at a stable level up to the end of the protocol (Day 35). This early phase of acute kidney injury (AKI), also described in mice models [75,110], was rapidly followed by infiltration of inflammatory cells in renal interstitium [111]. At day 10 after the beginning of the protocol, all biological parameters returned to basal levels reflecting an attempt of PTEC regeneration [112]. However, the presence of DNA adducts as well as the development of an inflammatory environment could interfere with PTEC attempting to regenerate. At day 35, AA-treated rats displayed foci of severe tubular atrophy surrounded by interstitial fibrosis (Figure 4), d tubular proteinuria, NAG, α-GST, LAP and NEP enzymuria have been shown to be enhanced, again characterizing this later phase of chronic kidney injury (CKD) [109].

Most of AAN cases that are reported in the literature describe patients with chronic kidney failure characterized by a typical interstitial fibrosis. Symptoms associated to AA nephrotoxicity can be detected years after cessation of herbal intake, resulting in delayed diagnosis and treatment. Nevertheless, few reported cases detail acute toxicity of AA [29,31,113] confirming the biphasic evolution described experimentally by Lebeau et al. Biopsies of these patients revealed interstitial nephritis, interstitial inflammatory infiltrate and evidence of acute tubular necrosis [29,31,113]. As experimentally described, majority of acute human cases reported by Yang [31] (*n* = 13) presented a progressive decrease of their renal function and five of them had reach the terminal stage of CKD at the end of the seven years follow-up.

#### 5.5.2. Cytotoxicity

##### DNA Adducts

As mentioned above, AA intoxication is strongly associated with the development of urothelial abnormalities as observed in AA-intoxicated patients all over the world [5,8,9,67] and confirmed in AAN experimental models [16,68,69,70,71]. In this regard, metabolic activation of AA to species forming DNA adducts is an important step for AA-induced malignant transformation. The major AA-DNA adducts found in AAN animal models and in AA-intoxicated patients were identified as 7-(deoxyadenosine-*N^6^*-yl) aristolactam I (dA-AAI), 7-(deoxyguanosin-*N^2^*-yl) aristolactam I (dG-AAI) and 7-(deoxyadenosine-*N^6^*-yl) aristolactam II (dA-AAII). Among them, dA-AAI has been found to be the most persistent AA-DNA adduct and constitutes a mutagenic lesion leading to an excess of A:T → T:A transversions [8,59,62,114]. The highest fraction of these transversions occurs in the kidney and in the bladder [98]. These specific mutations are retrieved at high frequency in codon 61 of the H-*ras* protooncogene in tumors induced by AAI in rodent models [115]. In AAN patients, an overexpression of p53 protein was observed suggesting a mutation in the tumor suppressor gene; *p53* [4]. In 2004, this mutation was identified as a specific AAG to TAG transversion in codon 139 (Lys → Stop) of exon 5 in the *p53* gene [116]. Interestingly, the same neighboring bases were observed in the mutated base adenine in codon 139 of the *p53* gene and in codon 61 of the H-*ras* gene suggesting a sequence specific mechanism during mutation induction [116]. Later, it has been described that A:T → T:A transversions constitute 58% of *p53* sequence changes found in UUC linked to AA exposure while it represents less than 2% in UUC patients with no suspected exposure to AA [117]. Moreover, these mutations described in AA-induced UUC have been found to be almost exclusively positioned on the non-transcribed strand which is rather unique hallmark because in other human cancers, A:T → T:A transversions do not present this pattern [59,117,118,119]. Specifically, mutational hotspots were observed at codons 131 and 179 and at the splice acceptor splice site for intron 6 [120]. Mutations in these sites had never been described in UUC and appear to be uniquely associated with AA exposure. Another recent study reported an unusually high prevalence of G → T transversions in the *p53* binding site in UUC of non-smoking AA-intoxicated women in Belgium (*n* = 5). The authors proposed these G → T transversions as a complementary signature mutation for AA intoxication [121].

##### Oxidative Stress

While the carcinogenicity of AA has been well investigated and documented, the mechanisms by which AA exert cytotoxic effects are poorly characterized. A few in vitro studies described that cells treated with AA led to generation of high amounts of reactive oxygen and nitrogen species (ROS/RNS) [122,123,124,125]. Oxidative stress therefore constitutes the primary triggering event of AA cytotoxicity and could be responsible for related DNA damage through activation of the MEK/ERK1/2 signaling pathway and depletion of intracellular glutathione (GSH) [122] resulting in cell cycle arrest in G2/M phase [123]. Treatment of the cells with antioxidants showed cytoprotective effects by reducing AA-induced ROS and genotoxicity, indicating that AA may induce DNA damage through oxidative stress [122,124]. Oxidative stress has also been described in in vivo models. In this regard, Pozdzick et al. showed that AA tubulotoxicity resulted in defective activation of antioxidative enzymes and mitochondrial damage in a rat model of AAN [112]. In C3H/He mice, Li et al. [126] demonstrated a reduction of renal antioxidant capacity in kidneys affected by AA, thereby suggesting that oxidative stress is involved in AAN. Their results indeed demonstrated a significant increase of the intra-renal concentrations of methylglyoxal (MGO), a highly cytotoxic compound that binds to proteins and forms *N*^ε^-(carboxymethyl)lysine (CML), an advanced glycation end product observed in the renal tubules by immunohistochemistry. Moreover, GSH levels were significantly decreased, along with a reduced intra-renal antioxidant capacity. Interestingly, a later study conducted by Huang et al. [127] reported an increased activity of renal semicarbazide-sensitive amine oxidase (SSAO), a key enzyme involved in MGO generation activity, in AA-treated mice and demonstrated the beneficial effect of MGO scavenging by metformin, which reduced nephrotoxicity. Therefore, AA-induced oxidative stress represents a key feature that could be considered as a potential target in view of therapeutic strategies. Recently, our group published an experimental study in which we confirmed that AA-intoxicated mice displayed significant increases in Nox2 mRNA expression and in plasma and urinary hydrogen peroxide concentrations, both markers of oxidative stress. Nox2 is widely expressed in the kidney and is indeed a major source of ROS. Moreover, oral l-Arginine treatment to AA-intoxicated mice was found to improve renal function and structure. These changes were associated to significant decreases in *Nox2* mRNA expression and in production of ROS along with an increase in antioxidant, superoxide dismutase (SOD), concentrations. All these observations led us to propose that maintaining NO bioavailability was beneficial for reducing ROS production [110].

##### Apoptosis

Apoptosis has been shown to be involved in the process of AA-induced tubular atrophy but the detailed mechanism for AA to induce apoptosis of renal tubular cells still needs investigation. It is now clear that AA treatment induce apoptosis in vitro as observed in cultured murine PTEC (mProx24) [128], in renal epithelial cells from pig (LLC-PK_1_) [78], in Mardin-Darby canine kidney (MDCK) cells [129] and in human PTEC (HK-2) [124]. A rapid rise in intracellular Ca^2+^ concentration has been described [129,130], which in turn determines endoplasmic reticulum and mitochondria stress [129], resulting in release of cytochrome *c* [130], caspases activation and finally apoptosis. Moreover, apoptosis and release of the cytochrome *c* were also observed in vivo in rat models [112,131] as well as in a mice model developed by our group where AA treatment was shown to increase expression of activated caspase 3. l-Arginine treatment to AA-intoxicated mice allowed to reduce this increase of expression thereby limiting apoptosis [110]. Finally, null mice for p53 displayed protection against AAN development allowing to consider that activation of the p53 pathway participate in the process of AA-induced apoptosis through dephosphorylation of STAT-3 [132]. Recently, p53 pathway has been shown to be inhibited by female hormone 17β-estradiol thereby reducing renal tubular injury in a mouse model of acute AAN [133]. Apoptosis is now considered as the major cell death pathway in AAN pathogenesis.

#### 5.5.3. Inflammation

Interstitial inflammation is a key factor in the progression to CKD. As an early phase of interstitial inflammation was observed following proximal tubular necrosis and preceding interstitial fibrosis [109,134], investigation and characterization of this inflammatory phase were undertaken. Indeed, it was postulated that the onset of an immune response could constitute the bridge between the two processes. In 1994, Depierreux had already mentioned the presence of a lymphocytic infiltration in biopsies of Belgian AAN patients [2]. Later, Pozdzik et al. described the kinetics of evolution of the inflammatory response in a rat model of AAN [111]. A massive interstitial infiltration of activated monocytes/macrophages and cytotoxic CD8^+^ and CD4^+^ T lymphocytes was shown around necrotic tubules only after three days of AA intoxication. It was proposed that the damaged PTEC could be responsible for this mononuclear cell influx into the renal interstitium. At Day 35 of the protocol, inflammatory infiltrates were still present, suggesting crucial interactions between tubular cells and immune cells. Finally, histological analyses of human AAN cases also revealed that inflammatory cells are preferentially present in the interstitium of the medullary rays and in the outer medulla even in terminal stages [135].

#### 5.5.4. Vascular and Tubular Compartments in AAN: The Egg or the Chicken?

Up to now, the PTEC have always been reported to be the primary target of AA-induced toxicity [109,112]. However, only few studies have addressed the involvement of the vascular network in AAN pathophysiology [2,30,110,136]. In this regard, Depierreux and colleagues were the first to raise the issue about the vascular network in AAN pathology [2] by describing a thickening of the walls of interlobular and afferent arterioles due to swelling of endothelial cells in human kidney biopsies from AA-intoxicated patients. Therefore, they proposed that primary lesions could occur in the vessel walls, then inducing tubular destruction [2,137]. In contrast, in a later study, Cosyns did not observe any vascular alterations in female New Zealand white rabbits injected intraperitoneally with a mixture of AAI and AAII during 17 and 21 months [71]. It was therefore concluded that vascular lesions observed in AA-intoxicated patients were not induced by AA but were more likely a late consequence of advanced kidney disease. Since then, other investigations have demonstrated a dramatic decrease in peritubular capillaries especially in the fibrotic areas in human renal biopsies as well as in experimental models [30,135,138]. In a rat model of AAN, the reduction of peritubular capillaries network was associated to a decreased expression of vascular endothelial growth factor (VEGF) and to an increased expression of HIF-1α thereby suggesting that ischemia and hypoxia are important factors contributing to AAN progression [138]. Similar data were also reported by Wen et al. in a rat model of AAN. Interestingly, they also described an imbalance between the vasoactive factors with a reduced NO production occurring along with an increase of mRNA and protein expression of endothelin-1 (ET-1) [136].

**Figure 5:**
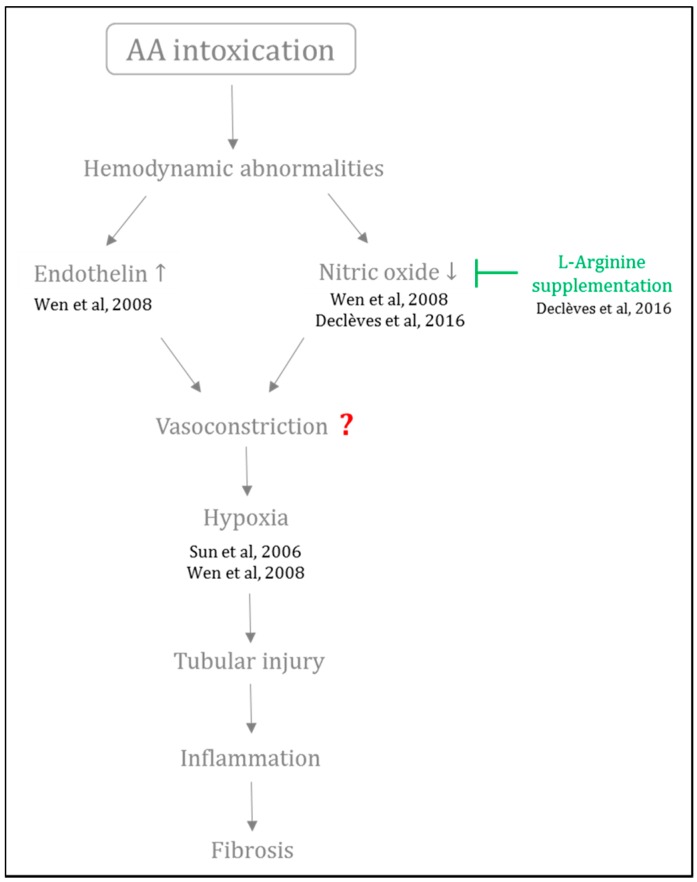
Involvement of hemodynamic abnormalities in progression of AAN. Following AA intoxication, hemodynamic abnormalities happen such as reduced renal nitric oxide bioavailability and increased renal endothelin. This imbalance between vasoactive agents could possibly lead to vasoconstriction and therefore renal hypoxia. All these events could contribute to tubular injury and to further progression to fibrogenesis characterizing chronic kidney disease.

Since the kidney has high energy demand, this organ is very susceptible to further compromise of vascular perfusion and oxygenation [139]. Although it is well known that injury of renal microvasculature, endothelial cell activation as well as imbalance between vasoactive substances widely contributes to the progression to CKD, these mechanisms are poorly investigated in AAN pathogenesis. In this regard, our group first investigated NO involvement in a mouse model of AAN [110,140]. We first confirmed the imbalance between vasoactive agents as observed by the decreased NO bioavailability in mice intoxicated with AA, only five days after the beginning of the AA intoxication. This effect was associated with a decrease of the renal function, massive necrosis of PTEC, increased inflammation and oxidative stress. We then demonstrated that restoring renal NO bioavailability by l-Arginine supplementation reduced oxidative stress, improved renal function and preserved renal structure [110]. These data may lead to consider that impairment of vasoactive mediators such as NO constitutes an early event of the disease, suggesting that impairment of the vascular compartment contributes to AAN pathogenesis. Although the precise sequence of molecular events that result in imbalance between the vasoactive substances as well as its consequences have not been completely elucidated, Figure 5 aims to summarize available data in the literature on this topic.

#### 5.5.5. Fibrosis

**Figure 6:**
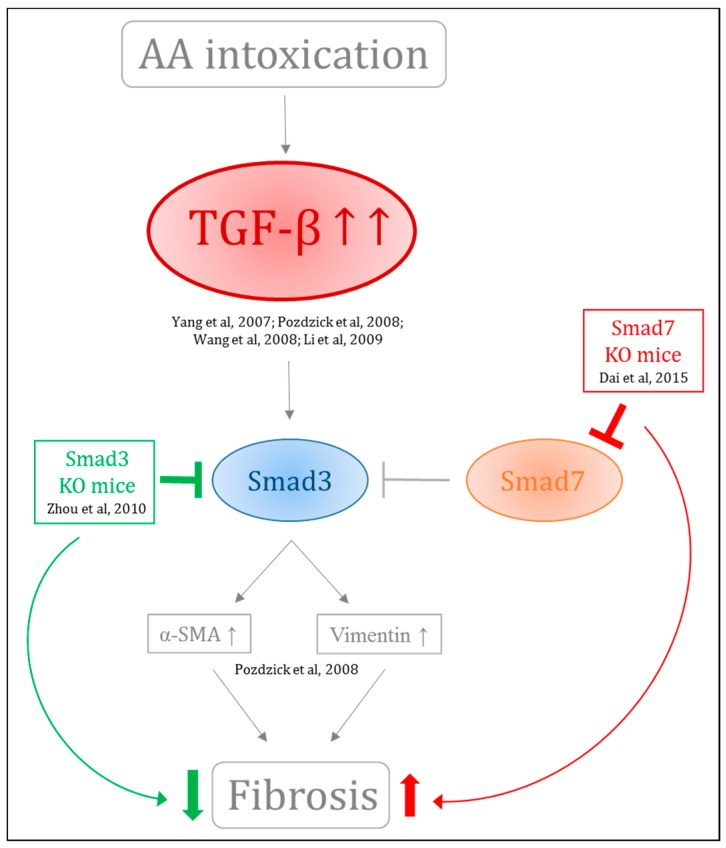
Involvement of TGF-β/Smad pathway in AA-induced fibrogenesis. TGF-β is overexpressed in AAN contributing to fibrogenesis. Downstream, Smad3 has emerged as a key factor tightly linked to matrix accumulation and deletion of Smad3 protects against several kidney disease. Smad7 negatively regulate Smad3 and KO mice for Smad7 intoxicated with AA presented enhanced progression of renal injury.

Tubulointerstitial injury characterized by significant fibrosis and tubular atrophy is considered as a final common pathway leading to ESRD. Irrespective of the nature of the initial renal injury, the degree of tubular and interstitial damages correlates well with the decline of the renal function and long-term prognosis. Renal fibrosis is the final common process defining CKD and is characterized by progressive tissue scarring leading to glomerulosclerosis and tubulointerstitial fibrosis and thereby constitutes a prominent feature of AAN. Pozdzik et al. reported that vimentin and α-smooth muscle actin-positive cells accumulated in the renal interstitium along with an overexpression of transforming growth factor-β (TGF-β) in rats intoxicated for 35 days with AA. TGF-β is the major driver of matrix synthesis, inhibition of matrix degradation and stimulator of myofibroblast activation [112]. Other groups have demonstrated its upregulation in AAN [30,111,112,141,142]. Molecular events involved in TGF-β overexpression are still unclear. However, more is known regarding the downstream pathway of TGF-β, the Smad protein family. Indeed, Smad3 has emerged as a key factor that has been tightly linked to matrix accumulation and deletion of Smad3 has been demonstrated to protect against several kidney disease, including AAN [143]. By contrast, KO mice for Smad7 intoxicated with AA exhibited enhanced progression of renal injury. This is of particular interest when keeping in mind that Smad7 negatively regulates Smad3. Moreover, restored renal Smad7 in the same KO mice resulted in protection against chronic AAN [144] (Figure 6). Involvement of bone morphogenetic protein-7 (BMP-7, a member of the TGF-β superfamily) in fibrogenesis was also investigated in vitro and in vivo. However, treatment with rhBMP-7 did not show any beneficial effect in AA-treated HK-2 cells and rats [145]. Recently, protein phosphatase magnesium-dependent 1A (PPM1A) has been demonstrated to exhibit Smad2/3 phosphatase activity. AA intoxication in mice resulted in decreased PPM1A expression initiating production of profibrotic factors [146]. Recently, activated platelet derived growth factor receptorβ (PDGFRβ)+ perivascular cells have been recognized as another main source of scar-associated kidney myofibroblasts. The inhibition of *p*-Smad2/3 signaling pathway by neutralizing anti-TGF-β antibody (1D11) administered during the acute phase of the rat model of AAN significantly reduced the score of acute tubular necrosis and interstitial inflammation, but also the extent of peritubular capillaritis and of PDGFRβ+ pericytes derived myofibroblasts accumulation [147].

Besides members of the TGF-β pathway, other factors were also investigated. Blockade of the renin-angiotensin system either with an angiotensin converting enzyme inhibitor alone or combined with antagonist of the angiotensin receptor 1 did not influence progression of fibrosis in a rat model of AAN. Thus, the pathways leading to interstitial fibrosis seem to be independent of the renin-angiotensin system in AAN model [134]. On the other hand, circulating transgene-derived hepatocyte growth factor has been shown to attenuate fibrosis without preventing tubular degeneration in a mice model of AAN [128]. Finally, inhibition of macrophages accumulation in AA-treated mice resulted in improved renal function and attenuation of interstitial fibrosis and renal inflammation therefore highlighting the crucial role of macrophages in production of TGF-β leading to renal fibrosis [148].

## 6. Conclusions

Today, the term “aristolochic acid nephropathy” is used to include any form of toxic interstitial nephropathy that is caused either by the ingestion of plants containing AA as part of traditional phytotherapies (formerly known as “Chinese herbs nephropathy”), or by the environmental contaminants in food (Balkan endemic nephropathy). Since its discovery in the early 1990s, many advances in understanding AAN pathogenesis have been made thanks to experimental in vitro and in vivo models. However, considerable challenges remain since no effective treatment has been found until now. In this review, we aimed to summarize data available in the literature about underlying pathophysiological mechanisms leading to AAN development with a particular emphasis on the vascular compartment as well as the imbalance between vasoactive factors that are often forgotten in AAN. Indeed, it is now greatly recognized in the literature that altered renal hemodynamics widely contribute to ongoing hypoxia and to the development from AKI to CKD. Therefore, we postulate that renal microvasculature injury and imbalance between endothelial vasoactive agents could contribute to renal dysfunction and, therefore, fibrosis in AAN. It therefore constitutes a critical point in AAN pathophysiology.

Finally, the transition of AKI to CKD has an important significance clinically because patients surviving an episode of AKI present a significant risk of progression to CKD [139,149,150]. However, the mechanisms by which AKI might initiate the onset of CKD have not been fully defined and better understanding of these pathophysiological mechanisms could lead to new biomarkers discovery as well as new therapeutic strategies to prevent and treat AKI or impede progression to CKD. In this regard, animal models of AAN could represent a useful tool and provide important insight into the underlying mechanisms of AKI-to-CKD transition.

## Figures and Tables

**Table 1:** Summary of reported cases of AA intoxications found in the literature.

Year of Publication	Authors	Country	Numb of Cases	Purpose of AA Ingestion *Suspected Aristolochia* Species
1993	Vanherweghem et al. [1]	Belgium	9	Slimming pills containing Chinese herbs. *Aristolochia fangchi*
1996	Peña et al. [22]	Spain	1	Infusion made with a mixture of herbs. *Aristolochia pistolochia*
1997	Tanaka et al. [35]	Japan	1	Health food for atopic dermatitis.
1998	Stengel and Jones [23]	France	2	Slimming pills containing Chinese herbs.
1998	Vanherweghem et al. [21]	Belgium	100	Slimming pills containing Chinese herbs. *Aristolochia fangchi*
1999	Lord et al. [24]	UK	2	Herbal preparation for treatment of eczema. *Aristolochia manshuriensis*
1999	Lee et al. [33]	Taiwan	1	Chinese herbal medicine for peripheral extremities weakness and numbness.
2000	Meyer et al. [26]	USA	1	Chinese herbal medicine for pain relief.
2000	Tanaka et al. [36]	Japan	2	Not described. *Aristolochia manshuriensis*
2000	Yang et al. [32]	Taiwan	12	Chinese herbal medicine for weight control, nutritional supplements, treatment of arthralgia, hypertension or hepatitis.
2001	Krumme et al. [25]	Germany	1	Chinese herbal medicine for hyperuricaemia.
2001	Chen et al. [28]	China	58	Not described.
2004	Lo et al. [29]	China	1	Chinese herbal medicine as a “tonic herbal remedy”.
2004	Lee et al. [39]	Korea	1	Chinese herbs mixture for slimming purposes.
2004	Kazama et al. [37]	Japan	1	Chinese herbal medicine for sterility.
2006	Hong et al. [34]	Taiwan	1	Chinese herbal medicines for “health improvement”.
2007	Yang et al. [30]	China	8	Chinese herb “Guanmutong” *Aristolochia manshuriensis*.
2011	Chau et al. [27]	Australia	1	Chinese herbal medicine to treat psoriasis.
2012	Yang et al. [31]	China	300	Not described.
2013	Michl et al. [38]	Bangladesh		Remedies for snake bites, sexual problems, gastric problems, “tonic remedy”. *Aristolochia indica*
